# Neurovascular Unit in Chronic Pain

**DOI:** 10.1155/2013/648268

**Published:** 2013-06-05

**Authors:** Beatrice Mihaela Radu, Placido Bramanti, Francesco Osculati, Maria-Luisa Flonta, Mihai Radu, Giuseppe Bertini, Paolo Francesco Fabene

**Affiliations:** ^1^Department of Neurological, Neuropsychological, Morphological and Movement Sciences, Section of Anatomy and Histology, University of Verona, Strada Le Grazie 8, 37134 Verona, Italy; ^2^Department of Anatomy, Animal Physiology and Biophysics, Faculty of Biology, University of Bucharest, 050095 Bucharest, Romania; ^3^IRCCS Centro Neurolesi “Bonino-Pulejo”, 98124 Messina, Italy; ^4^Department of Life and Environmental Physics, “Horia Hulubei” National Institute for Physics and Nuclear Engineering, 077125 Bucharest-Magurele, Romania

## Abstract

Chronic pain is a debilitating condition with major socioeconomic impact, whose neurobiological basis is still not clear. An involvement of the neurovascular unit (NVU) has been recently proposed. In particular, the blood-brain barrier (BBB) and blood-spinal cord barrier (BSCB), two NVU key players, may be affected during the development of chronic pain; in particular, transient permeabilization of the barrier is suggested by several inflammatory- and nerve-injury-based pain models, and we argue that the clarification of molecular BBB/BSCB permeabilization events will shed new light in understanding chronic pain mechanisms. Possible biases in experiments supporting this theory and its translational potentials are discussed. Moving beyond an exclusive focus on the role of the endothelium, we propose that our understanding of the mechanisms subserving chronic pain will benefit from the extension of research efforts to the NVU as a whole. In this view, the available evidence on the interaction between analgesic drugs and the NVU is here reviewed. Chronic pain comorbidities, such as neuroinflammatory and neurodegenerative diseases, are also discussed in view of NVU changes, together with innovative pharmacological solutions targeting NVU components in chronic pain treatment.

## 1. Introduction

According to the International Association for the Study of Pain (IASP), pain is an unpleasant sensory and emotional experience associated with actual or potential tissue damage, or described in terms of such damage [1].

Chronic pain onset can be sudden or slow and progressive, varies in intensity from mild to severe, and its end cannot be predicted. The diagnosis of chronic pain requires that the condition lasts longer than 3–6 months. Chronic pain can be a debilitating condition with potentially devastating impact on the quality of life [2]. It occurs in a wide variety of conditions, including peripheral neuropathy, stump pain, phantom pain, complex regional pain syndrome, central pain, polymyalgia rheumatica, fibromyalgia, pain of psychological origin, and epilepsy. The recently revised taxonomy includes several new conditions, such as chronic paroxysmal hemicrania: remitting form, hemicrania continua, postlumbar puncture headache, and so forth [1].

According to a report released in June 2011 by the Institute of Medicine of the National Academies, chronic pain affects about 100 million American adults—more than the total affected by heart disease, cancer, and diabetes combined [3]. The 2010 Patient Protection and Affordable Care Act required the Department of Health and Human Services of the United States of America to consider pain as a public health problem.

A 2006 study in 15 European countries and Israel indicates that chronic pain of moderate to severe intensity occurs in 19% of adult Europeans, seriously affecting the quality of their social and working lives [4]. A more recent evaluation of chronic pain in the European Union reports an even higher impact on the general adult population, with an average prevalence of 27%, similar to the that of other common chronic conditions [5].

Understanding the biological, cognitive, and psychological underpinnings of chronic pain represents a major research challenge. From a neurobiological standpoint, the cellular and molecular communication between the central nervous system (CNS) parenchyma and the circulating mediators of the immune and inflammatory response is at the core of such challenge. Indeed, an increasingly compelling body of evidence highlights a major role for the role of nonneuronal cells and diffusible mediators in the functional state of the brain, including neuronal excitability. The concept is captured in the term “neurovascular unit” (NVU), an ensemble of cellular and noncellular players (neurons, endothelial cells, glial cells, pericytes, the extracellular matrix, immune cells, inflammatory mediators) which form an integrated functional unit [6, 7].

In the context of the NVU, an obviously crucial role is played by the blood-brain barrier (BBB) and of the blood-spinal cord barrier (BSCB), both in general and with respect to the pathophysiology of chronic pain.

The purpose of this review is to explore the role played in the establishment and maintenance of chronic pain by the NVU, emphasizing (but not limited to) BBB and/or BSCB permeabilization phenomena. Chronic pain has a significant prevalence in neurodegenerative and neuroinflammatory pathologies, and BBB/BSCB permeabilization is discussed in this extended context. Finally, novel strategies targeting the NVU are considered for chronic pain relief. 

## 2. BBB and BSCB in the Neurovascular Unit

The importance of a full understanding of BBB/BSCB function is emphasized by its well-known role in regulating paracellular and transcellular drug transport, thus preventing or allowing CNS-acting drugs for chronic pain relief to reach their intended target [8]. In addition, there is a possibility that BBB/BSCB permeability may be altered in association with the development of chronic pain [9–12]. 

### 2.1. Anatomical Structure of Blood-Brain Barrier and Blood-Spinal Cord Barrier

The BBB is the regulating interface between circulating blood and brain parenchyma. Endothelial cells of brain capillaries, unlike those of the peripheral circulation, are characterized by the absence of cell membrane fenestrations, the presence of tight junctions, having a high number of cytosolic mitochondria, and minimal pinocytotic activity [7]. As an exception, the so-called circumventricular organs (CVOs) do possess fenestrated vasculature. In particular, secretory CVOs (median eminence and neurohypophysis) present a higher vascular permeability for low-molecular-mass tracers compared to sensory CVOs (organum vasculosum of lamina terminalis, subfornical organ, and area postrema) [13].

The surface area of the BBB, depending on the anatomical region, is between 150 and 200 cm^2^/g of tissue, resulting in a total area for blood-brain exchange between 12 and 18 m^2^ for the average human adult [14].

A functional equivalent of the BBB is the blood-spinal cord barrier (BSCB), constituted by nonfenestrated endothelial cells, basement membrane, pericytes, and astrocytic feet processes [15]. Several aspects distinguish BSCB from BBB, such as the glycogen deposits in the superficial vessels of the spinal cord [16], increased permeability to tracers and cytokines [17–19], decreased expression of tight-junction proteins and adherens junction proteins [20]. Such differences should be taken into account when these barriers are targeted for chronic pain treatment.

### 2.2. Mechanisms of Transport through Blood-Brain Barrier

The BBB has low passive permeability to many essential water soluble nutrients and metabolites required by the nervous tissue. However, in healthy conditions, the BBB shows temporary increases in permeability, allowing access to nutrients and oxygen. Since no brain cell is farther than about 15 *μ*m from a capillary [21], drugs and other solutes can rapidly reach all neurons and glial cell bodies, once the BBB has been crossed. Exchange of small organic compounds between blood and brain is regulated by plasma membrane transporters working either in the blood-to-brain direction, the brain-to-blood direction, or both. The directionality of transport is set by the subcellular location of the transport system (blood-facing or brain-facing membrane of the endothelial cells) and by the transport mechanism [8]. Several transport pathways have been identified in the BBB, such as (i) passive diffusion into brain of lipid soluble molecules (e.g., oxygen and carbon dioxide); (ii) ATP-binding cassette transporters (ABC-transporter, see below) efflux (P-glycoprotein (P-gp), multidrug resistance protein (MRP) 1–6, and breast cancer resistance protein transporters (BCRP)); (iii) solute carriers—SLC (transporters of glucose, amino acids, nucleosides, monocarboxylic acids, thyroid hormone, organic anions, organic cations, amine, and choline); (iv) transcytosis of macromolecules by receptor-mediated or adsorptive-mediated mechanism (transport of transferrins, lipoproteins, glycosylated proteins, IgG, insulin, leptin, tumor necrosis factor-alpha (TNF-*α*), EGF, LIF, cationised albumin, cell penetrating peptides); and (v) mononuclear leukocyte migration [22, 23]. In this review, particular attention will be devoted to ABC transporters with regard to chronic pain and BBB, as the majority of the analgesics are substrates for these transporters, especially for P-gp transporter [24–28]. 

The endothelial cells of capillary vessels play a major role in BBB physiology. The flattened cells present a luminal and an abluminal surface, separated by a 300–500 nm thick cytoplasm in human brain microvessels [29]. The tight junctions (TJs) connecting adjacent cells represent the most significant BBB structure and serve a dual purpose. On one hand, by sealing the intercellular space, they control the paracellular transport pathway (“gate function”). On the other hand, they effectively subdivide the membrane into two distinct functional domains (“fence function”) [30]. The endothelial cell polarization arises in particular from the differential expression of specific transporter proteins on either surface. The TJ-associated membrane proteins comprise occludin, tricellulin (also called marvelD2), cingulin, claudins (CL-1, CL-3, CL-5), junction-associated molecules of the immunoglobulin superfamily (JAMs), zona occludens proteins (ZO-1, ZO-2, ZO-3), 7H6, and AF-6 [7, 31, 32]. Signaling pathways involved in TJs regulation include G-proteins, serine-, threonine- and tyrosine-kinases, extra- and intracellular calcium levels, cAMP levels, proteases, and cytokines, and all these pathways share the modulation of cytoskeletal elements and the connection of TJs' transmembrane molecules to the cytoskeleton [31].

In pathological states, such as neurodegenerative diseases (including stroke, multiple sclerosis, rheumatoid arthritis, and AIDS dementia) or neuroinflammation, BBB has an uncontrolled and prolonged increase in permeability that results in vasogenic edema and leakage of neurotoxic plasma constituents [33].

### 2.3. ABC Transporters in BBB and BSCB

ABC transporters represent the largest family of transmembrane proteins. Upon binding ATP, these proteins translocate a wide variety of substrates across extra- and intracellular membranes, including metabolic products, lipids and sterols, and drugs [34]. Tight junctions and ABC transporters expressed in the brain and spinal cord endothelial capillaries represent the major “guardians” of the transport through BBB/BSCB endothelium. ABC transporters, such as P-gp, MRP 1–6, and BCRP, are expressed in the barriers endothelium both in humans and rodents [27].

It is presumed that only efflux transporters located on the luminal (apical) side of the endothelium can restrict drug uptake into the brain [25]. However, transport balance (influx/efflux) is dramatically affected by pathological stressors, such as *status epilepticus* and neurodegenerative diseases [47–49], and has been suggested to be also partly modified in inflammatory pain [26, 36, 37]. P-gp and BCRP expression in the BBB is regulated during early inflammatory stages by TNF-*α* and IL-1β [50].

Table 1 summarizes some of the links established so far between ABC transporters in BBB/BSCB and chronic pain.

In conclusion, ABC transporters appear to play an important role in inflammatory pain and in analgesia (opioids or nonsteroidal anti-inflammatory drugs). Knocking out genes encoding ABC transporters has consequences in inflammatory pain or analgesic profile. For example, knocking out the gene encoding for MRP4 increases inflammatory pain threshold [45] and knocking out the gene encoding for MRP3 alters morphine pharmacokinetics [44]. Therefore, these transporters, in particular P-gp, represent key molecules that might contribute to BBB/BSCB permeabilization induced by inflammation-like stimuli in various pain syndromes [26, 36, 37].

## 3. Cross Talk between NVU Partners in Chronic Pain

*In vitro* and *in vivo* animal studies have confirmed NVU cellular crosstalk in inflammation-induced hyperalgesia or nerve injury models and results can be extrapolated to chronic pain.

### 3.1. Glia-Neuron Interactions

Glia are significantly activated in response to trauma, ischemia, and invading pathogens by means of cytokine release (IL-1β, TNF-*α*) and may contribute to the maintenance of chronic pain [51, 52]. In addition to proinflammatory cytokine release at the peripheral site of injury, release also takes place in the CNS (spinal cord, brainstem, and forebrain) [53–55]. Released cytokines together with activated glia have been proved to influence and modulate neurons in the trigeminal nucleus region in a trigeminal model of inflammatory hyperalgesia [56]. On the other hand, different signaling pathways mediate IL-1β actions in hippocampal neurons compared to astrocytes [57].

Glia activation within the CNS has been suggested to maintain the pain sensation, even after the original injury or inflammation has healed, and convert it into chronic pain by altering neuronal excitability [58]. In a peripheral nerve injury pain model, the inhibition of microglia after four weeks from nerve injury normalized the pain threshold, while removing the inhibitor immediately restored pain-related phenomena [52].

### 3.2. Microglia-Astrocytes Interactions

Both *in vitro* and *in vivo* data provide clues on how the crosstalk between microglia and astrocytes may play a role in chronic pain maintenance [59–63]. The activation of microglia has been shown to cause astrocytic activation, with a delay of about 4 days [54, 64]. Preventing microglial activation (and subsequent astrocyte activation) inhibits hyperalgesia or allodynia [59, 61]. Once the astrocytes are activated, inhibiting microglia has no effect on pain [59, 60]. On the other hand, brain astrocytes can be activated in response to peripheral nerve injury without prior microglia differentiation [65]. A dialogue between microglia expressing IL-18 and astrocytes expressing its receptor (IL-18R) was suggested to be important in tolerance to morphine analgesia, by means of a P2X7R/IL-18/D-serine/N-methyl-D-aspartate receptor (NMDAR)/PKC*γ*-mediated signaling pathway [62], but also for tactile allodynia after nerve injury [66].

Increased monocyte chemotactic protein 3 (MCP-3, known as CCL7) expression associated with IL-6-dependent epigenetic modification at the MCP-3 promoter after nerve injury, mostly in spinal astrocytes, may serve to facilitate astrocyte-microglia interaction in the spinal cord and could play a critical role in the neuropathic pain-like state [63].

Some studies suggest the importance of the triad neuron-astrocyte-microglia in physiological and pathological inflammatory states [67]. 

### 3.3. Astrocyte-Endothelial Cell Interactions

Astroglial-endothelial signalling is altered under pathological conditions, such as infection, inflammation, stroke, or trauma, leading to BBB opening [6]. The coupling between the abluminal capillary cell membrane and the surrounding glial end-foot processes is reduced in pathological conditions [68, 69]. Stimulation of astrocytes, in coculture with brain endothelial cells, with 5-hydroxytryptamine (5-HT) generated a pronounced increase in intracellular Ca^2+^ release in the presence of inflammatory or pain-mediating activators, such as substance P, calcitonin gene-related peptide (CGRP), lipopolysaccharide (LPS), or leptin [70]. Mu-opioid agonists inhibit the enhanced intracellular Ca^2+^ responses in inflammatory-activated astrocytes cocultured with brain endothelial cells [70]. Overexpression of endothelin-1 in astrocytes, but not in endothelial cells, ameliorates inflammatory pain response after formalin injection [71]. The role played in chronic pain development by *in vivo* endothelial-astrocyte interaction at the barrier has not been investigated yet.

### 3.4. Pericyte-Endothelial Cell Interactions

*In vivo* studies in wild-type mice have shown that pericytes are more numerous in the brain than in the spinal cord [72]. Whereas brain regions such as the neocortex, hippocampus, and caudate nucleus show almost uniform presence of pericytes, the spinal cord shows significantly nonuniform distributions along the rostrocaudal extent, with the thoracic region being richer in pericytes, but with no more than 70% of brain levels. This reduced number of pericytes in the spinal cord correlates with (i) a higher BSCB permeability, as probed by fluorescent dextran and (ii) a diminished expression of tight junction proteins ZO-1, occluding, and claudin-5. Compared to wild-type mice, in *Pdgfrβ* ^F7/F7^ pericyte-deficient mice, pericytes are reduced more in spinal cord capillaries, leading to BSCB disruption to serum proteins. ZO-1 and occludin are also reduced, and the accumulation in motor neurons of cytotoxic thrombin and fibrin leads to motor neuron loss [72]. In another pericyte-deficient model, the *Pdgfrβ* ^ret/ret^ mouse, an increase in BBB permeability to water and to a range of low- as well as high-molecular-mass tracers has been shown [73]. Pericytes express MRP1, MRP4, and MRP5 transporters, which might imply a role played by these cells in regulating xenobiotic transport through the BBB [74].

Abnormal interactions between pericytes and endothelial cells have been implicated in a number of human pathological conditions, including tumor angiogenesis, diabetic microangiopathy, ectopic tissue calcification, and stroke and dementia syndrome CADASIL [75]. In pathological conditions implying BBB damage, such as stroke, hypoxia, and traumatic brain injury, the pericytes migrate away from brain microvessels wall and it seems to have an important role in neurovascular unit repair [76–78].

Morphine potentiates endothelial-pericyte interaction via platelet-derived growth factor-BB (PDGF-BB)/PDGF receptor-β (PDGFR-β) signaling and promotes tumor angiogenesis, pericyte recruitment, and coverage of tumor vessels [79]. The role of pericyte-endothelial cell interaction in chronic pain development and the role of pericytes during BBB disruption are still open topics.

## 4. BBB and BSCB in Chronic Pain: “To Be or Not to Be” Permeabilized/Disrupted

### 4.1. Acute/Chronic Pain Induces Changes at the BBB and BSCB Level

Does chronic pain cause BBB/BSCB permeabilization or disruption? The variety and complexity of the clinical conditions that involve chronic pain make a simple answer impossible, and studies on animal models of acute/chronic pain provide controversial responses to this hypothesis.

In the literature, the terms BBB “opening,” “leakage,” and “breakdown” are often used interchangeably, but more caution should be paid, when choosing between them [80]. A distinction should be made between BBB “permeabilization” and BBB “disruption” in experimental animal models. For the purpose of this review, the term BBB permeabilization refers to leukocytic recruitment associated with increased endothelial permeability, with no tight junction opening or altered efflux transport. As BBB “opening” or “permeabilization” is a physiological phenomenon, it should be reserved to transient processes [80]. On the other hand, we consider the BBB “disrupted” if Evans blue (EB) or albumins are extravasated into brain or spinal cord parenchyma. BBB “disruption” or “breakdown” represents a long-term opening associated with often-irreversible phenomena [80–82].

#### 4.1.1. BSCB Permeabilization in Neuropathic Pain and Disruption in Chronic Pain Animal Models

Peripheral nerve injury produced by either sciatic nerve constriction or selective transection (peroneal and tibial nerve branches, but not sural branch) causes a transient increase in BSCB permeability in the lumbar and thoracic spinal cord, peaking about 24–48 h. after injury and returning to normal levels after 7 days, as assessed by EB dye or horseradish peroxidase accumulation in the parenchyma [10]. BSCB permeability was also increased 24 hours after electrical stimulation of the sciatic nerve at intensity sufficient to activate C-fibers, but not A-fibers, or after capsaicin application on the sciatic nerve [10].

Partial sciatic nerve ligation in rats, a model of neuropathic pain, triggers an increase of BSCB permeability in the lumbar, but not in the thoracic, spinal cord to tracers of different size (e.g., EB, sodium fluorescein), which was prominent between day 3 and day 7, stayed significant for at least 4 weeks after injury, and returned to normal levels after 2 months [11]. Contrasting results on BSCB permeability in extralumbar spinal cord regions (e.g., thoracic) [10, 11] could likely be attributed to EB protocol differences.

Plasma proteins (IgG and fibronectin) immunopositive deposits in the ipsilateral side of the spinal parenchyma and downregulation of tight junction proteins (ZO-1, occludin-1, and caveolin-1) in isolated microvessels of the spinal cord were found 3 days after injury [11]. BSCB permeabilization occurs independently of the activation of resident microglial cells, EB extravasation being present while a microglial inhibitor minocycline is infused intrathecally from day 0 to day 7 [11]. Additionally, it was shown that the administration in rats of high doses of IL-1β (intravenous) impairs BSCB disruption, while TGF-β1 and IL-10 (intrathecal) shut down the openings in BSCB [11].

In a recent study of perispinal inflammation induced by applying the toll-like receptor (TLR)-2 agonist zymosan to the dorsal dural surface of the L1/L2 spinal cord, the lack of BSCB permeabilization was inferred from the lack of serum proteins in the spinal parenchyma 24 h after surgery [12]. No immunohistological evidence of T-cell or Mac-1-positive macrophages crossing into the parenchyma was found, but ATF-3 (a transcription factor that is also a sensitive indicator of neuronal injury) expression was observed in the dorsal horn of the same spinal cord segments after 1 day [12]. Thus, inflammatory signals are indeed transduced across the BSCB at the site of the inflammatory stimulus, within a 400–500 *μ*m radius. Astrocyte activation and gliosis are significantly increased in the superficial dorsal horn 1–7 days after surgery, with a transient recovery after 14 days, while resident microglia cells show a steady increase in staining density within the superficial dorsal horn beginning 1 day after surgery [12]. 

Neuropathic pain induced by L4 spinal nerve lesions in animal models is accompanied by astrocyte activation and albumin leakage, revealing BSCB disruption more prominent in the gray matter of the lesioned side compared to the contralateral in both dorsal and ventral horns [83]. Inflammatory events and changes in astrocyte and microglia reactivity at the spinal level in response to injury or disease are important processes that can initiate pain hypersensitivity [84, 85]. Studies conducted in a T-cell-deficient Rag1-null adult mouse have shown that T-cell infiltration and activation in the dorsal horn of the spinal cord following peripheral nerve injury contribute to the evolution of neuropathic pain-like hypersensitivity [86]. Most likely, the T-cell infiltration into the spinal cord is higher than normal in the nerve-injured animals, a fact that may be correlated with an increase in BSCB permeability.

BSCB permeabilization is a delayed event with respect to the initial injury and has a transient character. Studies addressing the role of the endothelium in BSCB disruption have been carried out, but the inclusion of the NVU as a whole is needed [87]. While the activation of glia may be important for the development of chronic pain, it is still unclear if the activation is required for BSCB disruption or if the two phenomena are independent. Peripheral inflammation or nerve injury in animal models induces astrocytes and microglia activation in the spinal cord [52, 88–91], but in these studies evidence regarding BSCB permeabilization is not available. In this view, new approaches connecting glia activation to BSCB opening would be very useful.

#### 4.1.2. BBB Permeabilization in Animal Models of Inflammatory Pain

Inflammation induced by an intraplantar injection of *λ*-carrageenan into the rat hindpaw causes increased brain uptake (*in situ* brain perfusion) of [^14^C]sucrose at 1, 3, 6 and 48 h after injection [92]. In the same study, Western blot analysis on isolated cerebral microvessels indicated a transitory increase in ZO-1 expression (increase after 1–6 h, returned to control after 12 h.) and a reduction in occludin expression (after 1, 3, 6, 12, and 48 h) [92]. These expression patterns indicate increased BBB permeability and suggest a link with the development of inflammatory pain. In another study devoted to inflammatory pain, [^14^C]sucrose *in situ* brain uptake, [^3^H]  *in situ* cerebral flow, and Western blot analysis (occludin, ZO-1, CL-1, and actin expression) were performed 1 h after formalin injection, 3 h after *λ*-carrageenan injection and 3 days after complete Freund's adjuvant (CFA) injection, and BBB permeabilization was observed [93].

In a rat model of inflammatory pain (injection of CFA into the plantar hindpaw), significant edema formation and hyperalgesia were observed 72 h. after treatment, together with significant increases in brain sucrose uptake. Expression of the transmembrane TJ proteins occludin, claudin-3 and -5, and junction adhesion molecule-1 (JAM-1) significantly changed 24–72 h after CFA injection, as proved by Western blotting [9] and confocal microscopy [94].

The induction of peripheral inflammatory pain through the injection of *λ*-carrageenan was associated with increased BBB permeability in a study that showed, by means of SDS-PAGE/Western blot analysis, a significant change in the relative amounts of oligomeric, dimeric, and monomeric occludin isoforms in BBB endothelial cells, presumably promoted by the disruption of disulfide-bonded occludin oligomeric assemblies [95].

Expression of organic anion-transporting polypeptide 1a4 (Oatp1a4) is upregulated after 3 h exposure to *λ*-carrageenan; the upregulation is prevented by diclofenac, suggesting the implication of acute/chronic inflammatory pain [36]. This modulation of BBB permeability in inflammatory pain appears to be controlled by the TGF-beta/activin receptor-like kinase-5 (ALK5) signaling pathway [96]. *λ*-carrageenan-induced peripheral inflammatory pain generates increased [ ^14^C]sucrose and [ ^3^H]codeine *in situ* brain uptake, and rats pretreated (10 min before *λ*-carrageenan injection) with tempol, a pharmacological ROS scavenger, have an attenuated radiotracers uptake [97]. In the same study, other indirect pieces of evidence for BBB modulation have been presented consisting in increase of the nitrosylated proteins in isolated brain vessels extract.

 In a *λ*-carrageenan inflammatory pain model, unidirectional permeability coefficients for several selected brain regions (hypothalamus, cerebellum, midbrain, cerebrum, hippocampus, brainstem, and thalamus) were calculated. Three hours after *λ*-carrageenan injection, the BBB resulted in an increased permeability in cerebrum and brainstem; diclofenac administration reversed this effect [98]. Western blot analysis of occludin expression in the same brain regions, however, did not reveal any significant changes [98]. In conclusion, correlating occludin expression changes with BBB “permeabilization” is problematic on the basis of the available data.

Administration of EB, which readily binds to serum albumins, is “classically” employed to assess BBB integrity, since in normal conditions the dye should not be found in the brain parenchyma [10]. However, in order to be revealed by EB, BBB disruption must be of a substantial degree (e.g., ischemic stroke [99]), while inflammatory pain per se does not constitute sufficient stimulus [100]. Inflammatory pain is more likely related to BBB permeabilization, as suggested by [^14^C]sucrose transport through the BBB using *in situ* brain perfusion [9]. 

Despite the valuable information contained in the above described studies, there are several experimental pitfalls to be considered. First, only indirect pieces of evidences are available in support of the idea of BBB permeabilization in inflammatory or chronic inflammatory pain. It is difficult to assess BBB permeabilization based on changes in TJ protein expression in an homogenate of isolated brain capillaries or to expand results from *in situ* brain perfusion with radioactive tracers to the BBB permeabilization. Another problem is that relatively short experimental durations (such as 24–72 h) are considered equivalent to a “chronic” pain state [9], while similar experiments on BSCB permeabilization were carried out over a significantly longer time scale (1 week–2 months) [10–12]. More consistent studies, based on *in vivo* brain uptake of Evans blue or [^14^C]sucrose should be done in order to prove BBB permeability changes. Alternative *in vivo* methods, such as intravital microscopy [101] or nuclear imaging of radioisotope-labeled leukocytes [72], are still unexplored in the field of chronic pain. A regional brain mapping of BBB permeabilization from the initial acute pain induction to the late chronic pain phase would be of significant use. In any case, clinical translation of the results obtained with experimental inflammatory pain models is still far from accomplished.

**Figure 1:**
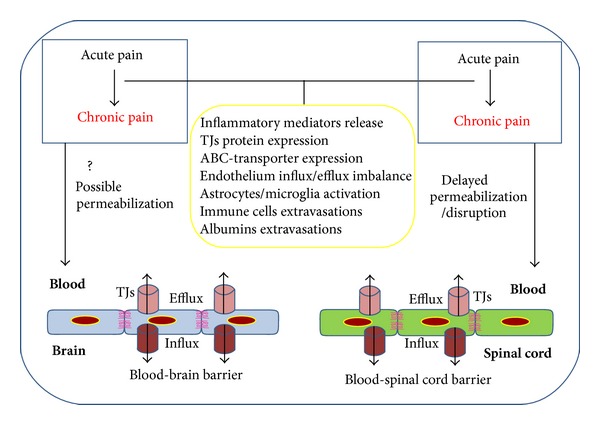
Acute pain occurs in a first step as a result of peripheral injury and/or inflammation. Chronic pain appears as a delayed event associated with permeabilization or brain/spinal cord capillary endothelium disruption. Different processes, such as inflammatory mediator release, changes in TJs protein and ABC transporters expression, activation of microglia and/or astrocytes, immune cells and albumin extravasation, may occur independently or in an “orchestrated” manner, and might contribute to the process of BBB/BSCB permeabilization or disruption.

Possible changes in BBB and/or BSCB permeability as a result of acute and chronic pain are shown in Figure 1.

### 4.2. Chronic Pain Treatments and NVU

Two major classes of analgesic drugs are currently in use for chronic pain treatment: opioids and nonsteroid anti-inflammatory drugs (NSAIDs). NSAIDs are used to treat chronic mild to moderate pain, while opioids are powerful analgesic agents used to treat moderate to severe chronic pain [102].

On the other hand, beside a wide range of adverse effects, long-term clinical administration of opioids (e.g., morphine) in chronic pain therapy is prevented by tolerance and dependence [102]. A classical dogma holds that agonist-induced *μ*-opioid receptor internalization contributes directly to functional receptor desensitization and opioid tolerance [103]. By contrast, other studies suggest that opioid receptor internalization can reduce opioid tolerance *in vivo* (reviewed by [103]). Beside neurons, other NVU players (e.g., glial cells, pericytes) have been considered to contribute to opioid tolerance development [62, 79, 104]. Endothelial cell lining represents the first “defence” to be crossed by opioids before interacting with CNS cells and therefore efflux alterations at this level are crucial in opioid tolerance.

In the *λ*-carrageenan rat model, acute inflammatory pain generates an increased functional expression and trafficking to membrane domains of endothelial efflux transporters (e.g., P-gp) in the BBB microvasculature [26, 37]. On the other hand, the same rats treated with morphine show reduced brain uptake of the drug due to increased P-gp activity [26]. Coadministration of cyclosporine A (P-gp inhibitor) with morphine in rats increased morphine transport through the BBB in a dose-dependent manner [26]. In the clinical practice, reducing tolerance to morphine by co-administration with cyclosporine A is unfeasible due to severe side effects (nephro- and neurotoxicity) [105, 106]. 

Chronic morphine treatment induced an increase in the expression of interleukin (IL)-18 by microglia, IL-18 receptor (IL-18R) by astrocytes, and protein kinase C*γ* (PKC*γ*) by neurons in the spinal dorsal horn. The results were interpreted by the authors as signs of a complex glia-neuron dialogue in the process of developing tolerance to morphine [62]. Morphine also potentiates endothelial-pericyte interaction via PDGF-BB/PDGFR-β signaling [79]. Morphine upregulates sphingolipid ceramide (in spinal astrocytes and microglia, but not in neurons) and spinal sphingosine-1-phosphate [104]. In turn, sphingosine-1-phosphate modulates spinal glial function, increasing the production of glial-related proinflammatory cytokines, in particular TNF-*α*, IL-1β, and IL6 [104].

Another major line of chronic pain treatment is represented by nonopioid analgesics such as NSAIDs. These drugs have several side effects, the most important being the risk of serious upper gastrointestinal complications, including bleeding, ulcers, and perforation [102]. NSAIDs act on the descending pain control system, which includes the periaqueductal gray matter and rostral ventromedial region of the medulla, which are also targets for endogenous opioids. Therefore, repeated administration of NSAIDs (e.g., metamizol, lysine-acetylsalicylate, analgine, ketorolac, and xefocam) to rats induces tolerance to themselves and cross-tolerance to opioids [107, 108]. 

Studies suggest that NSAIDs interact in several different ways with the brain endothelium, either by reducing edema and BBB/BSCB permeabilization [109, 110] or by inhibiting endothelial ABC transporters (e.g., MRP1, MRP4) [42, 43]. Diclofenac attenuates edema and hyperalgesia induced by *λ*-carrageenan in the cerebral and brainstem regions [98]. Indomethacin, an inhibitor of cyclooxygenase (COX)-1 and COX-2, reduces BBB damage induced by intracerebral injection of TNF-*α* [109]. Pretreatment with p-chlorophenylalanine, indomethacin, ibuprofen, and nimodipine of rats with spinal cord injury, reduced edema formation, BSCB permeabilization, and blood flow [110]. Indomethacin was shown to be an inhibitor of MRP1 function [42] and indomethacin, indoprofen, ketoprofen, and flurbiprofen inhibit MRP4 [43]. Diclofenac is transported by BCRP, but not by P-gp [39].

## 5. Comorbidities of Chronic Pain with Neuroinflammatory and Neurodegenerative Diseases: Role of the Neurovascular Unit

**Figure 2:**
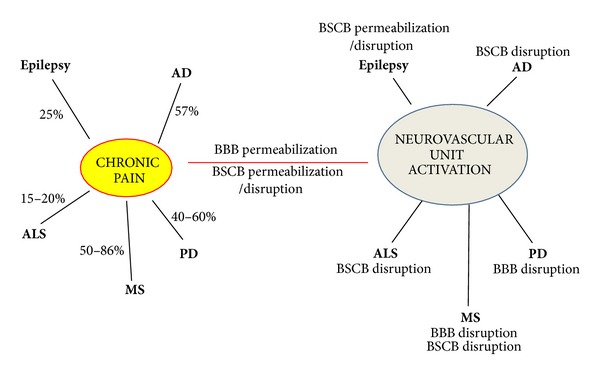
Similarities between chronic pain and NVU activation. Studies from the literature indicate that different NVU components are activated in a given pathology (e.g., epilepsy, Alzheimer's disease, Parkinson's disease, multiple sclerosis, amyotrophic lateral sclerosis, chronic pain) with a special focus on BBB/BSCB permeability alterations. Neuroinflammatory/neurodegenerative diseases are associated with chronic pain (see indicated percentages), but further studies are necessary to establish whether NVU activation may represent a “missing link” in the association. While the intrinsic mechanisms relating NVU activation, chronic pain, and neuroinflammatory/neurodegenerative disorders remain unclear, BBB/BSCB permeabilization appears to play a role.

Chronic pain has an extensive palette of comorbidities, but only neuroinflammatory and neurodegenerative diseases with known alterations of the NVU are here discussed (Figure 2). High prevalence of chronic pain can be observed in all these CNS pathologies. BBB/BSCB alterations in epilepsy, Alzheimer's disease, Parkinson's disease, multiple sclerosis, and amyotrophic lateral sclerosis will be briefly described, with regard to chronic pain syndromes. Understanding the exact role played by each pathology in permeabilizing/disrupting brain and spinal cord capillaries' endothelium is a crucial step in finding better therapeutic solutions.

### 5.1. Epilepsy and Chronic Pain

Epilepsy is a set of chronic neurological disorders characterized by abnormal, excessive, or hypersynchronous neuronal activity in the brain. The Epilepsy Comorbidities and Health (EPIC) Survey recently performed in the United States indicated that epilepsy is comorbid with several pain disorders, such as migraine, chronic pain, fibromyalgia, and neuropathic pain [111]. Additionally, the EPIC study indicated that chronic pain is prevalent in 25.4% of epileptic versus 17.7% of nonepileptic survey responders [111]. Chronic pain and fibromyalgia may be related to physical inactivity, which is more prevalent among adults with a history of epilepsy than among those without epilepsy [112].

The recent IASP taxonomy includes epilepsy in the list of generalized syndromes of chronic pain and includes chronic paroxysmal hemicrania—remitting form and hemicrania continua in the list of the chronic pain conditions [1]. Epilepsy and ictal epileptic headache share several pathophysiological mechanisms, such as (i) EEG abnormalities—lateralized or generalized, ipsilateral or contralateral, with focal theta activity or generalized spike-waves, and brief or longer-lasting episodes and (ii) headache and EEG anomalies resolve within minutes of i.v. antiepileptic medication administration [113]. The overlap between migraine and epilepsy may be partial or complete, not necessarily synchronous (preictal, ictal, or postictal), and in some cases the headache may represent the only ictal phenomenon [113]. In pediatrics studies, 3.1% of the patients suffered from idiopathic headache and idiopathic or cryptogenic epilepsy or unprovoked seizures [114]. The same study showed a strong association between migraine and epilepsy: in migraineurs the risk of epilepsy was 3.2 times higher when compared to tension-type headache, and children with epilepsy had a 4.5-fold increased risk of developing migraine than tension-type headache [114]. Postictal headache occurred in 41% of temporal lobe epilepsy patients, 40% of frontal lobe epilepsy patients, and 59% of occipital lobe epilepsy patients [115].

Several mechanisms have been proposed to explain comorbidity of epilepsy and chronic pain (such as that characterizing migraine), such as (i) the essential role of glutamate as a mediator of the hyperexcitability in both focal seizures and migraine, considering that seizure generation and spread are mediated by synaptically released glutamate acting on AMPA receptors, while triggering of cortical spreading depression depends on NMDA receptors and spread does not require synaptic transmission; (ii) mutations in genes for the membrane ion transport proteins CACNA1A (P/Q-type voltage-gated calcium channel), ATP1A2 (Na+-K+ ATPase), and SCN1A (voltage-gated sodium channel) [116]. 

Another important mechanism implied in chronic pain comorbidity with epilepsy is NVU activation. In this respect, brain endothelium seems to play an important role.

BBB disruption induces epileptiform activity [117–122]. We have previously shown that BBB leakage is induced by acute seizure activity but prevented by blockade of leukocyte-vascular adhesion, either with blocking antibodies or by genetically interfering with P-selectin glycoprotein ligand-1 (PSGL-1) function in mice [123]. Endothelial proinflammatory chemokines induce complex signal transduction pathways leading to integrin activation and controlling leukocyte recruitment, and therefore play a critical role in epileptogenesis [124, 125].

ABC transporters in the BBB are also affected in epilepsy. Shortly after *status epilepticus*, MRP1, MRP2, and BCRP are upregulated in astrocytes within several limbic structures, including hippocampus [47]. In chronic epileptic rats, these proteins are overexpressed in the parahippocampal cortex, specifically in blood vessels and astrocytes surrounding these vessels [47].

Whether transient BBB opening occurs during migraine attacks is controversial. Some magnetic resonance imaging studies have reported negative results [126, 127] while others have found indications of BBB leakage [128]. In migraine, indirect evidence for BBB permeabilization is provided by increased circulating levels of matrix metalloproteinases (MMPs) 2 [129] and 9 [130] that have been attributed to MMPs release from the extracellular matrix of the neurovascular unit.

### 5.2. Alzheimer's Disease and Chronic Pain

Alzheimer's disease (AD) is the most common cause of dementia. It is a neurodegenerative disorder characterized by synaptic and neuronal loss, by the accumulation in the extracellular matrix of beta-amyloid deposits, and by the presence of abnormal aggregates of microtubule-associated proteins, the so-called neurofibrillary tangles, in neuronal cell bodies.

Prevalence of pain in AD was estimated at 57% of all patients [131], although such assessment is complicated by two factors. First, pain processing may be altered in dementias [132, 133] including AD [134]. Second, the primary method for pain assessment is patient reporting [135], but pain affects cognitive function [136, 137] and cognitive function in turn affects pain [133], which makes pain assessment in AD very difficult. 

Astrocytes tend to localize around fibrillar amyloid plaques, suggesting that Aβ deposition is a potent trigger of astroglial activation in the AD brain [138]. Additionally, an increase in the number of IL-1 immunoreactive microglia associated with AD plaques has been shown [139]. A variety of biomarkers for microglial activation in AD have been proposed, such as chitotriosidase, CCL18 (pulmonary activation-regulated chemokine; PARC), YKL-40, CCL2 (monocyte chemoattractant protein 1; MCP-1), CD14, and neopterin [140].

Immunohistochemistry on postmortem human brains affected by AD or vascular dementia indicated an increased expression of CL-2, Cl-5, and CL-11 in neurons and of CL-2 and CL-11 in astrocytes and oligodendrocytes [141]. There is a strong relationship between neurodegeneration, cognitive decline, and BBB disruption in AD [142]. It was suggested that during neurodegeneration the receptor for advanced glycation end products (RAGE), which mediates transfer of amyloid-β to the brain through the endothelial cells, can be upregulated [143]. In AD transgenic mice, BBB alteration was proven to precede accumulation of senile plaques [144].

Neuroinflammation represents a crucial part in the pathogenesis of AD and other neurodegenerative diseases [145]. Inflammatory mediators, such as IL-1β, IL-6, TNF-*α*, IL-8, transforming growth factor-β (TGF-β), and macrophage inflammatory protein-1*α* (MIP-1*α*), are upregulated in AD [146].

### 5.3. Parkinson's Disease and Chronic Pain

Parkinson's disease (PD) is a degenerative disorder of the CNS, mainly characterized by loss of dopamine-generating cells in the substantia nigra. Prevalence of pain (musculoskeletal pain, neuritic or radicular pain, dystonia-associated pain, primary or central pain, and akathitic discomfort) in PD is estimated around 40–60% [147, 148]. See [149] for a comprehensive review of pain in PD.

Impairment of BBB function has been implicated in the pathogenesis of PD. Accumulation of verapamil (normally extruded from the brain by P-gp) in the brain of PD patients proves a dysfunction of BBB [150]. Injection of dopamine neurotoxin 6-hydroxydopamine (6OHDA, which produces Parkinson's-like dopaminergic neuron lesions) into the striatum of rats induced FITC-labeled albumin leakage in areas of the brain that are not protected by the BBB (e.g., the hypothalamus around the third ventricle and area postrema along the floor of the fourth ventricle) but no leakage in BBB-protected areas (e.g., ipsilateral parietal cortex or hippocampus, or into contralateral structures) [151]. The presence of neuroinflammatory markers, such as activated microglia or astrocytes, is also an important feature of PD [152]. Microglia activation and upregulation of inflammatory mediators can be induced by *α*-synuclein and contributes to PD pathogenesis [153]. On the other hand, astrocyte activation in PD is still under debate [152, 154–158]. In a recent study performed on aged c-rel^−/−^ mice developing PD-like degeneration of substantia nigra pars compacta, we observed a marked microglia activation in the substantia nigra pars compacta and striatum, but no GFAP-positive astrocyte activation [159].

### 5.4. Multiple Sclerosis and Chronic Pain

Multiple sclerosis (MS) is a chronic inflammatory disease of the CNS, which leads to demyelination, neurodegeneration, perivascular edema, and inflammatory infiltrates [160]. Prevalence of pain in MS is estimated around 50–86% [161, 162]. A recent classification based on pathophysiological mechanisms and response to treatment identified nine types of MS-related pain: trigeminal neuralgia and Lhermitte's phenomenon (paroxysmal neuropathic pain due to ectopic impulse generation along primary afferents), ongoing extremity pain (deafferentation pain secondary to lesion in the spinothalamocortical pathways), painful tonic spasms and spasticity pain (mixed pains secondary to lesions in the central motor pathways but mediated by muscle nociceptors), pain associated with optic neuritis (nerve trunk pain originating from nervi nervorum), musculoskeletal pains (nociceptive pain arising from postural abnormalities secondary to motor disorders), migraine (nociceptive pain favored by predisposing factors or secondary to midbrain lesions), and treatment-induced pains [163]. 

BBB disruption is an early event in the progression of MS, as proved by magnetic resonance imaging studies [164, 165]. Diapedesis of monocytes and subsequent trafficking of monocyte-derived macrophages into the brain are key mediators of demyelination and axonal damage in MS. Endothelin 1 (ET-1), its type B receptor (ET(B)) and endothelin-converting enzyme-1 (ECE-1) are mediators for monocyte diapedesis through the human BBB and play a key role in demyelination and axonal damage in MS [166]. In experimental models of MS, such as experimental autoimmune encephalomyelitis (EAE), BBB disruption is induced by T-cells in conjunction with antigen-presenting dendritic cells [167, 168], and monocytes [169]. MS lesions are often found in proximity to blood vessels [170], associated with loss of occludin and ZO-1 in the microvasculature [171–173]. Leukocyte extravasation through BBB is mediated by cytokines: TNF-*α*, IL-1B, and interferon-*γ* [174]. Infiltration of inflammatory cells are localized perivascularly, but can also be located in the CNS parenchyma. In acute inflammatory lesions, CD4^+^ and CD8^+^ T cells and B cells infiltrate the lesion site. Lesions at later MS stages show an abundance of macrophages with internalized myelin degradation products and reactive proliferating astrocytes [175]. Sodium channels contribute to activation of microglia and macrophages in EAE [176].

MS is also characterized by significant changes in the composition and dynamics of the BSCB [177]. CD3-positive T-cells accumulate within the dorsal horn in mice with EAE, early in the disease course when cold and tactile allodynia are observed [178]. BSCB disruption is greatest at disease onset, followed by inflammation and demyelination, indicating that increased BSCB permeability precedes the destructive inflammatory process [177]. A recent study showed that autoreactive T cells access CNS via the fifth lumbar spinal cord in EAE mouse model [179].

In an EAE model, a recent study suggested a signalling role for Wnt (a family of secreted signaling proteins) in MS-associated chronic pain pathogenesis, although only neurons and glial cells were examined [180]. On the other hand, Wnt signaling contributes to brain angiogenesis, BBB formation, influences vascular sprouting, remodelling, and arteriovenous specification by modulating the Notch pathway [181]. Therefore, further studies on Wnt signalling in brain microvasculature could bring new insights in to MS-related pain syndromes. 

### 5.5. Amyotrophic Lateral Sclerosis and Chronic Pain

Amyotrophic lateral sclerosis (ALS) is a chronic, progressive, and ultimately fatal neurodegenerative disease of motor neurons in the brain and spinal cord [182]. Prevalence of chronic pain (especially located at the arms level) in ALS is estimated around 15–20% [183, 184].

Increased permeability of the BSCB has been implicated in the pathogenesis of ALS [185]. Studies conducted in the ALS mouse model SOD1-G93A have shown BBB and BSCB disruption [186, 187], in areas of motor neuron degeneration (early and late ALS stages) [186] and capillary rupture in brainstem (early symptomatic ALS stage) [186]. Some studies indicate reduction in tight junction proteins (ZO-1, occluding, and claudin-5) before motor neuron loss, in presymptomatic ALS stages [188], while other data point out the reduction in tight junctions proteins (ZO-1 and occludin) and basement membrane protein agrin in symptomatic ALS stages [187]. Therefore, it is still controversial if the BBB/BSCB disruption is the cause or the consequence of ALS development. In SOD1-G93A mice, an increase was detected in mRNA and protein levels for P-gp and BCRP at the level of capillary endothelium in several regions, such as whole spinal cord, cerebral cortex, and cerebellum [189]. Additionally, the transport activity of P-gp and BCRP increased with ALS progression in spinal cord and cerebral cortex capillaries [189].

T lymphocytes are able to cross into the brain and spinal cord parenchyma, where they interact with resident microglia, inducing them to adopt either an M1 (cytotoxic) or M2 (protective) phenotype, depending on ALS stage [190]. Clinical studies evidenced perivascular and intraparenchymal CD4^+^ T-lymphocytes in the proximity of degenerating corticospinal tracts and ventral horns in two-thirds of ALS patients [191]. CD4^+^ T-lymphocytes slow disease progression, modify the microglial phenotypes, and extend survival [192, 193]. A potential mechanism behind the longer life expectancy may be mediated by the augmented secretion of IL-4 from mutant Cu^2+^/Zn^2+^ superoxide dismutase regulatory T lymphocytes that directly suppressed the toxic properties of microglia [193]. It was suggested that CD4^+^CD25^High^FoxP3^+^ regulatory T lymphocytes (Tregs) are neuroprotective and slow ALS progression [194].

## 6. Potential Strategies Targeting BBB or BSCB for Chronic Pain Relief

The molecular mechanisms of BBB/BSCB permeabilization due to chronic pain have yet to be clarified. Nevertheless, the barriers represent promising targets in designing new therapeutic strategies for chronic pain. Several approaches tested in preclinical and clinical studies, such as the use of Rho-kinase inhibitors, antiepileptic compounds, and statins, might turn out to be viable solutions in the future.

### 6.1. Rho-Kinase Inhibitor

Rho kinase (ROCK) is involved in various physiological functions, including cell motility, vasoconstriction, and neurite extension. ROCK inhibition reduces tissue-type plasminogen activator (t-PA)/plasminogen-mediated increase in permeability of *in vitro* models of the BBB [195]. Fasudil, a specific ROCK inhibitor, partly alleviates EAE-dependent damage by decreasing BBB and BSCB permeability [196]. In preclinical models of pain, fasudil (30 mg/kg) significantly attenuated mechanical allodynia in spinal-nerve ligation, chronic constriction injury, capsaicin-induced secondary mechanical hypersensitivity, sodium iodoacetate-induced pain, and capsaicin-induced acute flinching behaviors, but failed to attenuate or had only modest effects on inflammatory thermal hyperalgesia following carrageenan injection and mechanical allodynia following complete Freund's Adjuvant injection [197]. Fasudil also proved to be efficient in adjuvant-induced arthritis model (inflammatory arthritis model) and a monoiodoacetate-induced arthritis model (noninflammatory arthritis model) [198]. 

### 6.2. Antiepileptic Drugs

It is difficult to consider currently market-available antiepileptic drugs (AEDs) as an alternative for classical analgesics because of their side effects, potential drug interactions, and unsatisfactory efficacy (epilepsy resistance). Between 1990 and 2012, 16 new AEDs were approved, most of them developed using mechanism-unbiased anticonvulsant animal models [199]. In order to be attractive for the pharmaceutical industry, the future design of new AEDs must also include a potential in nonepileptic CNS disorders, such as bipolar disorder and neuropathic pain [199]. Resistance to AEDs is encountered in more than 40% of epileptic patients [25], probably due to upregulation of the efflux transporters in brain capillary endothelium [200]. 

Only three AEDs are currently approved by the Food and Drug Administration (FDA) and European Medicines Agency (EMA) for the treatment of neuropathic pain: carbamazepine (CBZ), gabapentin (GBP), and pregabalin (PGB), all of them considered first-line treatment options for several neuropathic pain conditions (reviewed by [199]). Randomized clinical trials in spinal cord injury-related pain indicate gabapentin and pregabalin as powerful analgesics [201]. Cochrane Library reports based on extended clinical trials indicate GBP, PGB, and lacosamide, but not valproic acid, to be efficient against neuropathic pain or fibromyalgia [202–205].

Levetiracetam (LEV) may constitute a novel approach for BBB protection [206]. Clinical studies have evidenced the effects of levetiracetam (LEV) in various pain conditions, such as postmastectomy pain syndrome, trigeminal neuralgia, chronic general or central pain in MS, lumbar radiculopathy, chronic daily headache, polyneuropathy, and central poststroke pain [207–212]. In a rat model of hypothermia-induced cortical dysplasia, LEV and topiramate were found to protect the BBB [212]. However, a recent clinical trial failed to reveal significant effects of LEV against spinal cord injury-related pain [213]. Despite LEV's protective properties on the BBB, clinical efficacy against chronic pain is still controversial.

### 6.3. Statins

Beside the well-known efficacy of statins (inhibitors of HMGCoA (3-hydroxy-3-methyl-glutaryl-coenzyme A) reductase) in lowering plasma cholesterol levels, these compounds show a large palette of pleiotropic effects. Statins can improve endothelial function (thereby regulating the BBB permeability), decrease the oxidative stress and inflammation, and generally have a beneficial effect on the immune system, central nervous system, and bone [214]. Some of these effects point out statins as good candidates for chronic pain treatment. *In vivo *preclinical tests showed that Atorvastatin (a lipophilic statin) restored the BBB permeability in mice fed with saturated fatty acids (which compromised BBB integrity) [215]. In primary human skeletal muscle myoblast cells, atorvastatin and rosuvastatin proved to be substrates for MRP1, MRP4, and MRP5 transporters [216]. 

An analgesic effect was revealed by hot-plate test for some statins [217]. Preclinical tests have been performed to evaluate statin efficacy in neuropathic pain. Daily administration of statin for two weeks completely prevented the development of mechanical allodynia and thermal hyperalgesia in a nerve injury model [218]. Such approaches provide promising results for considering statins as a possible future generation of drugs against chronic pain, especially for patients with dislipidemy.

## 7. Future Perspectives

General mechanisms of chronic pain onset, development, and maintenance still await clarification, and the particular relationship between chronic pain and NVU function is an especially complex issue. Whether permeabilization/disruption of the endothelial barrier in brain or spinal cord could be a cause and/or a consequence of chronic pain is an open topic. Clearly, a better knowledge of the neurovascular unit contribution to chronic pain physiopathology would be highly beneficial in the clinical practice, especially in view of pharmacological targeting of the NVU.

The use of currently available analgesics (opioids and NSAIDs, in particular) in chronic pain is limited by their side effects and by the induction of tolerance and/or dependence. In this review, we have described some aspects of the neurobiological mechanisms of chronic pain, with particular emphasis on NVU players' interactions, also in view of present and future treatments. Future strategies against chronic pain should take into account the essential role played by the neurovascular unit in the efficacy of analgesics in an effort to overcome the already-known problems.

As many neurodegenerative/neuroinflammatory pathologies are comorbid with chronic pain in a significant number of patients, the identification of dual-target therapeutic strategies should be considered a priority.

With the NVU as an increasingly relevant target for the treatment of chronic pain, development of immunologically based strategies for preventing BBB and/or BSCB permeabilization or disruption would also represent an opportunity. 

## Figures and Tables

**Table 1:** ABC transporters presence in BBB and BSCB and their potential role in pain.

ABC transporter (gene)	BBB	BSCB	Localization in brain capillary endothelium	Direction of efflux/influx	Implications in pain/analgesics or anti-inflammatory drugs versus ABC transporters
P-gp (ABCB1)	Yes [24]	Yes [35]	Luminal [25]	Blood [25]	There is an increased P-gp expression and dynamic redistribution between membrane domains of P-glycoprotein and caveolin-1 in peripheral inflammatory pain [26, 36, 37]. P-gp is involved in pain control with opioid analgesics [38]. Diclofenac is not transported by P-gp [39].

MRP1 (ABCC1)	Yes [24, 25, 40]	Yes [40]	Luminal [25] Abluminal [24]	Blood [25] Brain [24]	The nonsteroidal anti-inflammatory drug indomethacin, an efficient analgesic in some forms of trigeminal autonomic cephalalgias (e.g., paroxysmal hemicrania) [41], was proved to inhibit MRP1 function and expression in cancer cell lines [42]. Most probably indomethachin inhibits MRP1 in BBB. Diclofenac, rofecoxib, and celecoxib are poor inhibitors of MRP1 in HEK293 cells [43].

MRP2 (ABCC2)	Yes [24, 27]	Yes [35]	Luminal [25]	Blood [25]	Diclofenac is not transported by MRP2 [39].

MRP3 (ABCC3)	Yes [24]	?	Abluminal [24]	Brain [24]	Mice lacking MRP3 show altered morphine pharmacokinetics and morphine-6-glucuronide antinociception [44].

MRP4 (ABCC4)	Yes [24, 27]	?	Luminal [25] Abluminal [24, 25]	Blood [25] Brain [25]	Mice lacking MRP4 show increases in inflammatory pain threshold compared to wild-type mice [45]. MRP4 acts as a prostaglandin efflux transporter and is inhibited by nonsteroidal anti-inflammatory drugs (e.g., indomethacin, indoprofen, ketoprofen, and flurbiprofen) [43]. Diclofenac, rofecoxib, and celecoxib are poor inhibitors of MRP4 [43].

MRP5 (ABCC5)	Yes [24, 27]	?	Luminal [25] Abluminal [24]	Blood [25] Brain [24]	?

MRP6 (ABCC6)	Yes [24, 27]	Possible [46]	Abluminal [24]	Brain [24]	?

BCRP (ABCG2)	Yes [24]	Yes [35]	Luminal [25]	Blood [25]	Diclofenac, an analgesic mainly used against cancer- associated chronic pain, is efficiently transported by murine BCRP1 and moderately by human BCRP [39].
